# Patient-derived rectal cancer organoids—applications in basic and translational cancer research

**DOI:** 10.3389/fonc.2022.922430

**Published:** 2022-07-26

**Authors:** Yumeng Yan, Io Hong Cheong, Peizhan Chen, Xiaoguang Li, Xianli Wang, Hui Wang

**Affiliations:** ^1^ School of Public Health, Shanghai Jiao Tong University School of Medicine, Shanghai, China; ^2^ State Key Laboratory of Oncogenes and Related Genes, Center for Single-Cell Omics, School of Public Health, Shanghai Jiao Tong University School of Medicine, Shanghai, China

**Keywords:** rectal cancer, organoids, patient-derived, precision medicine, treatment prediction, tumor microenvironment, cancer modeling

## Abstract

Colorectal cancer (CRC) is one of the most commonly diagnosed cancers and among the leading causes of death in both men and women. Rectal cancer (RC) is particularly challenging compared with colon cancer as the treatment after diagnosis of RC is more complex on account of its narrow anatomical location in the pelvis adjacent to the urogenital organs. More and more existing studies have begun to refine the research on RC and colon cancer separately. Early diagnosis and multiple treatment strategies optimize outcomes for individual patients. However, the need for more accurate and precise models to facilitate RC research is underscored due to the heterogeneity of clinical response and morbidity interrelated with radical surgery. Organoids generated from biopsies of patients have developed as powerful models to recapitulate many aspects of their primary tissue, consisting of 3-D self-organizing structures, which shed great light on the applications in both biomedical and clinical research. As the preclinical research models for RC are usually confused with colon cancer, research on patient-derived RC organoid models enable personalized analysis of cancer pathobiology, organizational function, and tumor initiation and progression. In this review, we discuss the various applications of patient-derived RC organoids over the past two years in basic cancer biology and clinical translation, including sequencing analysis, drug screening, precision therapy practice, tumor microenvironment studies, and genetic engineering opportunities.

## Introduction

Cancer is a leading cause of noncommunicable disease deaths (9.9 million people annually) and has sparked a health crisis worldwide (1). Colorectal cancer (CRC) is the third most common type of cancer and second in terminology of cancer mortality. CRC includes colon cancer (CC) and rectal cancer (RC); 40% of CRC is represented by RC (2). Although early diagnosis and multiple treatment strategies optimize outcomes for individual patients, we still need a more accurate and precise model to facilitate cancer research, including predicting response to standard therapies, as large numbers of newly developed strategies fail in clinical trials but go well on cancer models (3).

Traditional immortalized two-dimensional cancer cell lines and patient-derived xenografts (PDX) have long been applied in CRC research (4–11). Several studies from Bardelli’s laboratory are focused on 151 established CRC lines (6) along with xenopatient-derived cell lines or xenolines (XLs) (7, 8). The authors later improve this platform to compare drug responses and better carry on genomic analysis. As a result, this system is able to detect CRC dependencies on kinases for which clinically approved drugs are available and to discern CRC cross-sensitivity to Olaparib and Oxaliplatin. However, the translation of the obtained results from laboratory to bedside is often hampered as cell lines fail to recapitulate primary tumor characteristics due to lack of heterogeneity, fewer cancer cells expand from the microenvironment, and experience loss of their primary polarity (12). Furthermore, different types of PDX models, such as murine (9) and zebrafish (10, 11) are developed to study CRC tumor initiation, progression, immune response, and response of novel strategies. The histology and genomic mutational patterns of their primary tissue are maximally retained in PDX (13). Yet, due to high costs and low success rates, their use in cancer research is limited. In addition, animal models have conquered some of these drawbacks, but they do not mirror human physiology, resulting in the high failure rate of new cancer strategies in the clinic (14).

Organoid technology bridges the gap between cell culture and animal models, providing *in vivo*-like conditions. In general, organoids involve most types of cells from primary organs and can recapitulate the key features, main structure, and tissue functions along with gene expression profiles of the organs from which they were derived. To date, organoid technology nowadays has shown great capacity in breaking down mechanisms associated with gastrointestinal cancer and improving patient outcomes as recently reviewed by Lau et al (15). The novel technology based on generating patients’ tumors from biopsies as patient-derived organoids (PDOs) constitutes a major breakthrough for the study of translational cancer research, such as studying tumor biology, discovering new biomarkers, drug screenings, and testing targeted personalized therapies. Furthermore, huge efforts have been made in CRC research using the technology of tumor-derived organoids. The success establishment rate could reach around 63% by using small amounts of starting material from tissue biopsy samples (16, 17). These cancer organoids can be accomplished in a few weeks with high efficiency, and most of them have inheritable stability after several generations.

More than one third of CRC is represented by RC (2). Although CC and RC are synonymously called CRC, as they share the same histological classification and characteristics (18), RC is particularly challenging compared with CC since treatment of the RC patient is usually more complex than that of the CC patient due to their differences in anatomical position (19). Some RC patients can avoid surgery if they respond completely to neoadjuvant chemoradiotherapy alone (20, 21), and others responding poorly to CRT require radical surgery (22). Therefore, it is promising to use a correct and specific research model for identifying patients’ response to treatment in order to minimize potential harm from overtreatment and enable personalized therapy of RC.

Despite the fact that RC treatment is more complicated by utilizing trimodal therapy consisting of neoadjuvant chemoradiation, surgical resection, and 5-fluorouracil-based chemotherapy (19), few efforts have been made to develop RC-specific research models—not to mention that one preclinical practice for treatment of RC has unexpectedly depended on CC cell lines (23). Cell lines are also established from RCs (SW837, Caco-2, etc) (24–26), but whether they were derived from the correct side of the rectum or from patients in the context of multimodal therapy is hard to prove. As for the PDX platform, the first study to establish a PDX model from RC patients’ biopsies before treatment is reported recently, supporting the translational suitability of the PDX platform that predicted the response of corresponding patients, and to identify cetuximab as a strategy to enhance the efficacy of 5-fluorouracil/radiotherapy (27).

As PDOs can be easily converted into PDX models and represent one of the models that comes closest to the primary patient’s tumor, it currently shows potential in high-throughput drug screening and personalized therapy testing. Whereas extensive research is available for CRC organoids, most studies have not distinguished CC organoids from RC. For instance, we can find 214 full text results from the past two years through searching “(Patient-derived organoid) AND (colorectal cancer)” in PubMed and 132 full text results by applying the MESH “Patient derived organoid AND Colorectal Cancer” with the same year range, whereas only 26 full text results were found with the same year range by using “(Patient-derived organoid) AND (rectal cancer)” and 14 full text results were found by applying the MESH “Patient derived organoid AND rectal Cancer.” Furthermore, we find that some CRC-associated research did not mention whether it used samples from CC or RC patients (**Table 1**), and some clinically derived CRC organoids or biobanks exclude tissue from RC patients and mainly focus on CC specimens (30, 38) with RC PDOs remaining an underexplored field. This may be because patients with RC are often irradiated prior to surgery or treatment, which can affect the efficacy of derived organoids to assess the effectiveness of subsequent treatment strategies. However, with the continuous improvement of technology, we are looking forward to seeing that research in the field of CRC can be more refined in the future, and research models specifically used for RC will continue to be applied. Recently, specific research or protocols on the organoid model of RC is gradually garnering attention from scientists in the last two years, and translational applications are springing up (16, 39–41)(**Table 2**). The translational application of the RC PDO model is also mentioned in some recent literature and systematic reviews (44, 46, 50, 52, 53). There is a need for standard procedures and methods to improve the reproducibility and stability during laboratory practice.

**Table 1:** Excluded records of the CRC cancer organoid studies, rectum organoid research and others.

Category	Reference or NCT number	Excluded reasons
CRC cancer organoid studies	(28)	CRC research with organoids generated from naive WT mice normal intestine without from rectal tumor.
	(29)	CRC research with organoids generated from naive WT mice intestine without from mice rectal tumor.
	(30)	Built biobank excluded tissue from RC patients.
	(31)	CRC Immuno-genomic research used samples collected both from CC and RC patients without distinguishing them.
	(32)	CRC research used PDO model only derived from right-sided colon tumors without from rectal tumor.
	NCT05304741, NCT04996355, NCT05384184, NCT04755907, NCT05183425, NCT04220242, NCT02732860, NCT04587128, NCT04279509, NCT05412706	These clinical CRC studies did not mention whether RC patients were involved in their studies.
	NCT05038358, NCT04896684	Clinical studies related to colon cancer patient-derived organoid.
Rectum organoid research	(33)	Normal Rectum research used adult normal mucosa organoids.
	(34)	Normal Rectum research used mouse Rectum Crypt organoids.
	NCT03874559	Rectal cancer study used malignant colonic organoids model.
Others	(35)	Review did not focus on RC PDOs models.
	(36)	Colonic inflammatory bowel disease research used PDO model without mentioning Rectal cancer and related PDO models.
	(37)	Research used colonic epithelial organoids without mentioning Rectal cancer and related PDO models.

**Table 2:** Summary characteristics of included publications (n = 21) and clinical studies (n = 5).

Reference or NCT number	Study type	Source & type of experimental model	Limitions	Main findings
(42)	Analysis of the mutational landscape using high-throughput sequencing technologies	Excised from mice with RC patient tumor xenograft *Ex vivo*	• Lack of data from post treatment human rectal cancer specimens. • Did not show accordance exists between organoid and biopsy data • Small sample size	Assessed for ST6GAL-1 protein with and without chemoradiation treatment on patient-derived xenograft and organoid models and identified ST6GAL-1 protein as a mediator for resistance to clinical chemoradiation therapy through restraining apoptosis.
(43)	Personalized medicine based on the testing of individual PDOs; Studying the tumor microenvironment with PDOs; Cancer modeling by genetic engineering of organoids	Biopsies from pre-CRT tumor and normal *In vivo* and *ex vivo*	• Lack of data from post treatment human rectal cancer specimens. • Did not mention about the type and number of cells seeded for organoid culture. • Lacked the success rate and cell composition of the established organoid.	Developed RC PDOs and primary stroma cells and identified that interleukin-1α (IL-1α) after irradiation polarizes cancer-associated fibroblasts toward the inflammatory phenotype together with triggering oxidative DNA damage; Displayed the impact factor in chemoradiotherapy resistance and disease progression.
(44)	Reviewing biomarkers and models used in RC	–	–	Reviewed published findings associated with biomarkers discovery and pre-clinical models (included RC PDOs) in RC.
(45)	Personalized medicine based on the testing of individual PDOs	Surgically or endoscopically resected tumor tissues of patients undergoing neoadjuvant therapy *Ex vivo*	• Organoid culture lacked microenvironmental regulation of tumor response. • Did not mention about the type and number of cells seeded for organoid culture. • Lacked the success rate and cell composition of the established organoid.	Analyzed radiosensitivity of PDOs and provided a readout predictive of neoadjuvant therapy for selecting patients who need pre-treatment.
(46)	Reviewing PDOs models for precision medicine	–	–	Evaluated the potential of PDO models (included RC PDOs and distinguished RC research) in predictive translational research.
(47)	Conducting clinical trial for translational research from bench to bedside; Personalized medicine based on the testing of individual PDOs	–	–	Started ACO/ARO/AIO-21 phase I trial to test the IL-1 receptor antagonist (IL-1 RA) anakinra combining with CRT therapy for RC based on previous achievement (43), which set up a great example for translational application from bench to bedside.
(38)	Personalized medicine based on the testing of individual PDOs	0.5×0.5×0.5 cm for surgically resected specimens and 1.5×0.2×0.2 cm for ultrasound-guided core-needle biopsy tissue *Ex vivo*	• Results need further validation in the prospective, randomized controlled study. • Organoids culture was in the absence of tumor microenvironment. • Lacked the purity and cell composition report of the established organoid.	The sensitivity, specificity, and accuracy of the RC PDOs for predicting chemotherapy regimens response were 63.33%, 94.12%, and 79.69%.
(48)	Analysis of the mutational landscape using high-throughput sequencing technologies; Personalized medicine based on the testing of individual PDOs	Biopsy samples Ex vivo	• Small sample size • Did not mention about the type and number of cells seeded for organoid culture. • Organoids culture was in the absence of tumor microenvironment. • Did not perform a drug sensitivity test.	Established a prediction model through a machine learning algorithm combining clinical and experimental radio response data; Radiation responses in clinic were positively correlated with the paired cultures.
(41)	Analysis of the mutational landscape using high-throughput sequencing technologies	Colon-endoscopic biopsy from participants accepted preoperative chemoradiotherapy (pCRT) Ex vivo	• Did not show accordance exists between organoid and biopsy data	High expression of VSTM2L reduced *γ*-H2AX expression in RC PDOs treated with CRT.
(49)	Analysis of the mutational landscape using high-throughput sequencing technologies	Biopsy samples Ex vivo	• Small sample size • Did not mention about the type and number of cells seeded for organoid culture. • Lacked the success rate and cell composition of the established organoid.	Developed RC PDOs to detect genes and pathways that participate in the radio-resistance of LARC by biological and bioinformatic analysis approaches; Identified cathepsin E (CTSE) that was negatively correlated with the radio-resistance in PDOs.
(50)	Reviewing PDOs models for precision medicine	–	–	Described CRT prediction value of organoids (included RC PDOs) for GI cancers.
(51)	Drug screening to develop novel treatment strategies; Personalized medicine based on the testing of individual PDOs	Resected specimens Ex vivo	• Small sample size • Did not mention about the type and number of cells seeded for organoid culture. • Lacked the purity and cell composition report of the established organoid.	Screened PDOs with a customized medium-throughput drug library consist of 33 single agents and three 5-FU-based drug combinations with Leucovorin (FLV), Oxaliplatin (FLOX), and SN-38 (FLIRI).
(52)	Reviewing pre-clinical models used in RC	–	–	Described different pre-clinical model (included PDOs) used in RC research.
(53)	Reviewing biomarkers and models used in RC	–	–	Reviewed published paper associated with potential biomarkers and cell-based models (included RC PDOs) to predict treatment response in RC.
(16)	Analysis of the mutational landscape using high-throughput sequencing technologies; Drug screening to develop novel treatment strategies; Personalized medicine based on the testing of individual PDOs	Tissue biopsies from patients with newly diagnosed LARC who were treatment-naive in a phase III clinical trial NCT02605265 Ex vivo	• Lacked the purity and cell composition report of the established organoid.	Established an organoid biobank with PDOs obtaining similar histological and genetic features of original tumors; identify the role of predicting LARC patient Chemoradiation responses in the clinic.
(54)	Drug screening to develop novel treatment strategies	7 rectal endoscopic biopsy and 1 colon cancer sample from low anterior resection *Ex vivo*	• Lacked the success rate and cell composition of the established organoid.	Butyrate could enhance the curative effect of radiotherapy while protecting the normal mucosa; Identified FOXO3A as a factor with non-responsive cases to butyrate in PDOs.
(27)	Personalized medicine based on the testing of individual PDOs	Endoscopic biopsies from 26 Stages 2 and 3 rectal cancer patients prior to receiving 5FU/RT *In vivo* and *ex vivo*	• Small sample size	Identified the ability of cetuximab to enhance RT effectiveness; Used PDOs to improve patient selection based on mutational profile. Success rate:90%
(55)	Analysis of the mutational landscape using high-throughput sequencing technologies	Endoscopic Biopsies from therapy-naïve rectal cancer patients *Ex vivo*	• Did not mention about the type and number of cells seeded for organoid culture. • Lacked the success rate and cell composition of the established organoid.	Compared the gene profiling of organoids derived from a normal rectum and rectal tumors and their responses to calcitriol; Identified rectal tumor organoid-specific genes associated with biosynthetic machinery, including those encoding the RNA polymerase II subunits POLR2H and POLR2J.
(56)	Personalized medicine based on the testing of individual PDOs	Did not mention *Ex vivo*	• Did not mention about the type and number of cells seeded for organoid culture. • Lacked the success rate and cell composition of the established organoid. • Lack details in culture methods • Small sample size	Similarly, two patient-derived organoid models containing relatively low AC expression were found to be comparatively more radiosensitive than three other models containing higher levels of AC.
(39)	Analysis of the mutational landscape using high-throughput sequencing technologies; Drug screening to develop novel treatment strategies; Personalized medicine based on the testing of individual PDOs; Investigation of intratumoral heterogeneity and tumor evolution	Endoscopic biopsies from pre- and post-treatment patient samples *In vivo* and *ex vivo*	• Need studies with larger populations to investigate the prediction value. • Did not mention about the type and number of cells seeded for organoid culture.	RC PDO cultures reserved architecture and molecular features of the original tumors and their in vitro responses to clinical treatment correlated with the outcomes of individual patients’ tumors; PDOs from patients with RC under multimodal therapy engraft into the rectal mucosa of mice, which indicating a success in vivo RC PDO model.
(40)	Protocols for RC PDO establishment	Surgery or biopsy *Ex vivo*	• Lack details in culture methods • Lacked the success rate and cell composition of the established organoid.	Developed protocols for establishing RC cancer organoids; performed high-throughput drug sensitivity testing.
NCT03577808	Personalized medicine based on the testing of individual PDOs;	Pre-treatment biopsies *Ex vivo*	–	Validation of Organoids Potential Use as a Companion Diagnostic in Predicting Neoadjuvant Chemoradiation Sensitivity in Locally Advanced Rectal Cancer
NCT05352165	Personalized medicine based on the testing of individual PDOs; Drug screening to develop novel treatment strategies;	Not mentioned	–	A Prospective Multicenter Randomized Controlled Trial of the Clinical Efficacy of Neoadjuvant Therapy Based on Organoids Drug Sensitivity Versus Empirical Neoadjuvant Therapy in the Treatment of Advanced Rectal Cancer.
NCT04371198	Determine the feasibility of establishing patient-derived organoids.	Pre-treatment rectal adenocarcinoma biopsies. *Ex vivo*	–	Accessing the feasibility of the Biospecimen Collection Protocol for establishing Patient-Derived Organoids for Rectal Cancer
NCT05401318	Personalized medicine based on the testing of individual PDOs; Drug screening to develop novel treatment strategies;	Fresh tumor samples from colon and rectal cancer patients *Ex vivo*	–	Accessing the prediction value of the PDOs and investigating the effect of Pre-treatment with cytotoxic agents which can induce cellular immunotherapy efficacy against solid tumors in PDOs
NCT04842006	Personalized medicine based on the testing of individual PDOs;	Not mentioned *Ex vivo*	–	Population distribution of PDO treatment response is compared to their corresponding clinical response by response MRI and pathological response.

**Figure 1:**
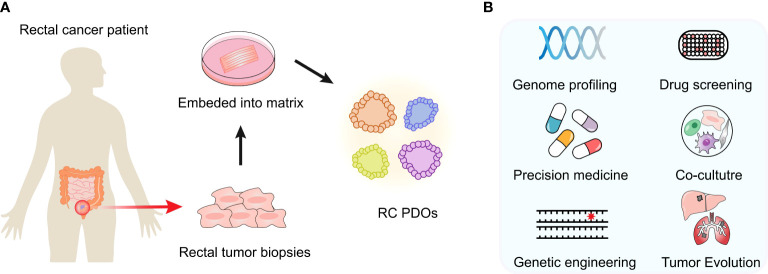
Developments and cutting-edge applications of the PDO model. **(A)** After an RC patient tumor’s surgery or biopsy, the tumor specimen is collected. PDOs can be generated in the laboratory and expanded to create sufficient material for biobank building and storage. Once established, these models are expanded in order to create sufficient material for storage and biobanking. **(B)** Multiple applications in vitro or ex vivo can be performed, such as genome profiling, drug screening, coculture experiments, etc.

In this review, we aim to review the latest in RC organoid development and its cutting-edge applications for basic or translational cancer research (**Figure 1**), including mutational analysis, drug screening, personalized medicine therapy, tumor heterogeneity, and microenvironment study as well as cancer modeling.

## Analysis of the mutational landscape using high-throughput sequencing technologies

Organoids over a period of continuous culture represent one of the *in vitro* models that comes closest to the primary tumor tissue, and they retain the function, histology, and genomic mutational patterns of the primary tumor, thereby preserving the heterogeneity of the origin tissue. By undergoing RC PDO sequencing, even low-frequency subclonal mutations can be detected and identified, and this gives scientists a hand in studying cancer pathobiology.

In the field of RC PDOs, two published articles in *Cell Stem Cell* and *Nature Medicine* (16, 39) illustrate the successful practice of RC organoids derived from patients, representing a major breakthrough in the precision medicine field. One group generated a biobank of 80 locally advanced RC PDOs from biopsy samples. Whole-exome sequencing was constructed in 18 culture lines, and the results displayed the mutational profile level of the corresponding tumor tissue. A 94.4% overlay between known cancer driver mutations and the TCGA data set was found in PDO samples. In 12 of 18 cases, they compared the genome-wide gene copy number variations of RC PDO cultures with that of primary tumors. The results displayed that the DNA copy number losses and gains of the original tissue were retained in PDOs. At the same time, WNT signaling was reported to represent the most related mutated genes pathway (88.9%), including APC (72.2%), FBXW7, TCF7L2, ARID1A, LRP5, and SOX9 mutation (16). Another group investigated the mutational fingerprint and larger landscape of molecular alterations in RC PDOs by undergoing the Food and Drug Administration–cleared tumor-profiling panel, MSK Integrated Mutation Profiling of Actionable Cancer Targets (MSK-IMPACT). The data displayed that 92% (range: 0.66–1.00) of the oncogenic mutation was retained in the paired PDO cultures compared with an 88% concordance (range: 0.62–1.00) reported of organoids from colon and colon metastases (with RCs excluded), and 77% of the clonal oncogenic mutations in the original tumors were also detected as clonal mutations in PDO cultures. The top mutations in the RC PDOs included the alterations of genes *APC*, tumor protein P53 (*TP53*)*, KRAS*, and F-Box and WD Repeat Domain Containing 7 (*FBXW7*) (39).

After a previous successful practice, more specific works are springing up. Costales-Carrera A et al. analyze the RNA-seq transcriptomes of six normal rectum or colonoscopy and rectal tumor organoid cultures by establishing a biobank of 50 organoids from therapy-naive RC patients. They confirm that these established RC organoids involve classic genetic alterations of sporadic CRC (APC, TP53, KRAS). Interestingly, some aberrant genes presented expression differences in RC and CC organoids. A cluster of genes regulating protein synthesis (ribosomal biogenesis, translation components, and regulation) was only expressed at a high level in rectal tumor organoids, indicating that CC and RC mainly differ in protein expression rather than the RNA level (55). In a new coclinical trial, Park et al. compares the result of PDTOs after irradiation with the individual patients’ response of radiotherapy. They used targeted next-generation sequencing analysis to check the ability of organoids to recapitulate the paired patients’ tissue and found that the match rate of KRAS, NRAS, and BRAF mutations in PDTOs could reach to 86.6%, 100%, and 100%, respectively. WNT signaling pathway–associated genes (including *APC* and *FBXW7*) were mutated in 68.4% of established cultures with identified *APC* (68.4%) and *FBXW7* (31.5%). But, there was a problem in that the sample size was small, and they did not mention whether these PDOs were derived from patients who were therapy-naïve or not (48).

To analyze relevance between gene expression and the radiosensitivity of PDOs, Lee et al. checked 27,685 genes and identified 1741 differentially expressed genes from radio-resistant (RR) and radio-sensitive (RS) organoids by RNA-seq. In RR organoids, up-regulation genes that encode the control protein include calcium-dependent interactions (ANXA2, S100A4), immune cell activation (CD55, IL18, RUNX3), receptor catabolic processes (NPC1, APOE, LGMN), and plasma membrane proteolysis (ADAM9, BACE1, CTSE). They found the enhanced expression of CTSE regulated by DNA methylation status in RR organoids, suggesting that CTSE could be applied as a biomarker for radio-responsiveness (49).

One group combined the results from PDX, RC organoids generated from xenograft mouse models, and RC cell lines and identified ST6GAL-1 protein as a mediator for resistance to clinical chemoradiation therapy through restraining apoptosis. The data might need further studies in a larger sample size system (42).

Another report mentions that the high expression of VSTM2L could be one of the bad factors leading to patient resistance to CRT through diminished *γ*-H_2_AX expression, but they all did not mention about the correlation between PDTO response and patient outcomes (41).

Overall, these studies confirm the feasibility that rectal organoids can be generated and cultured from the clinical patients’ specimens and the great potential of PDO libraries as powerful platforms for RC research *in vitro* since the gene expression profiles of PDOs are very close to the organs from which they were derived.

## Drug screening to develop novel treatment strategies

Some RC patients can avoid surgery if they respond completely to CRT alone (20, 21), whereas others responding poorly to CRT require radical surgery (22). So it is crucial to undergo drug screening to minimize potential harm from overtreatment and enable personalized therapy.

PDOs now represent the most precise and effective models for drug screening and research. To date, Yao et al. generated 80 organoid cultures derived from patients with locally advanced RC and tested their susceptibility to 5-FU, irinotecan, or radiation. These data support that RC PDOs can function as a biomarker to help identify therapy-sensitive patients and promote precision medicine (16). Another group further leveraged an RC PDO platform to test responses to treatment in the front line, including FOLFOX (5-FU, leucovorin, and oxaliplatin) or radiation. Particularly, the responses of RC PDOs were consistent with the progression-free survival of the patients (39). It is also reported that PDOs derived from RC patients are used as a system to access the effect of combined radiotherapy with the short-chain fatty acids (such as butyrate, propionate, and acetate) by regulating HDAC activity. They further identified FOXO3A as a factor with cases nonresponsive to butyrate in PDOs. These data displayed the advantages of PDOs for testing novel drugs and modulating drug response (54). Kryeziu K et al. tested the drug efficacy of a medium-throughput drug library comprising 33 single agents and three 5-FU-based drug combinations containing Leucovorin (FLV), Oxaliplatin (FLOX), and SN-38 (FLIRI) by screening RC PDOs. They applied an interdisciplinary method to analyze spatiotemporal pharmacogenomic heterogeneity in a recurrent patient with KRAS-mutated liver metastases from RC. Coclinical *ex vivo* analyzes of front-line neoadjuvant combination chemotherapy regimens in three subsequent liver resections simulate actual responses to treatment, including indications of acquired resistance to FLOX. This case sets a great example for clinical application except for the small sample size. It is promising to conduct practice based on more large samples for evaluating the potential of PDOs in precision medicine (51).

These studies provide evidence that the capacity of PDOs to discover novel strategies and undergo drug screening overcome the flaws of 2-D cancer cell lines and upgrade the success of newly developed drugs.

## Personalized medicine based on the testing of individual PDOs

RC is an ideal disease type to conduct precision medicine practice due to the different clinical responses of different patients and the subsequent tailored treatment. If the expected radiosensitivity is poor, patients may need more intensive chemotherapy application, and if the pretreatment worked well with the patients, they could avoid radical surgery. Accordingly, stable and reliable prediction tools play an important role in clinical practice for RC patients. It is reported that PDOs preserve high predictive value and the possibility to validate clinical biomarkers.

Ganesh et al. treated organoid culture from RC patients’ tumors of different stages with clinical chemotherapy (5-FU alone or with oxaliplatin together) or radiation and analyzed the corresponding clinical response of each patient. They revealed that the PDO predictions were correlated with the results from clinical patients after treatment. They also successfully used tumoroids to establish the endoluminal model as a feasible and reproducible *in vivo* platform of RC and invested its chemosensitivity. They found that the *in vivo* model could also recapitulate the clinical response to therapy such as 5-FU and FOLFOX (39). In a clinical phase III study, Yao et al. reported an LARC organoid biobank that could foresee the clinical response to 5-FU, irinotecan chemotherapy, irradiation, or combined treatment (16). They compared the radiosensitivity of organoids with clinical patients’ responses and found that 16 out of 17 patients with irradiation-sensitive organoids displayed positive response upon noadjuvant chemoradiation (NACR) treatment. Among the 64 patients whose organoids were resistant to irradiation, 42 of them had a poor response to NACR. They also exposed RCOs to 10 μM 5-Fu or 10 μM CPT-11. Twenty-seven patients with 5-Fu-sensitive organoids achieved a good response except for five poor response cases, and 38 of the 53 patients with 5-Fu–resistant PDOs had a poor response, and another 15 performed well. As for CPT-11, 32 sensitive PDOs of paired patients achieved a good response except for seven cases, and 27 of 34 patients with resistant organoids had a poor response. They, together, reveal that patients can acquire a good clinical response if their paired tumor organoids were sensitive to at least one of the three preceding treatments, which means that those with 5-Fu– or CPT-11–sensitive PDOs would probably benefit from 5-Fu or CPT-11 treatment alone although their PDOs were resistant to irradiation. Although PDOs generated by Yao et al. lack sources from patients of different stages compared with that from Ganesh et al., the data from these two groups could provide the evidence that the PDOs may enable tailored, personalized strategies with chemoradiation or chemotherapy and reduce overtreatment or toxicities for RC patients.

Later, Janakiraman et al. found that cetuximab has the potential to selectively sensitize the radiation effect with a KRAS mutational condition existing, and this group also detected cetuximab sensitivity gene impactors by establishing novel PDX and related PDO platforms from RC patients under pre-neoadjuvant therapy (27). Another observational coclinical study checked the standard combination of chemotherapy regimens in a PDO model generated from a patient with recurrent KRAS-mutated liver metastases from RC. They identified the SMAC mimetic as a unique therapeutic option in PDOs from the recurrent tumor tissue (51). Park et al. developed a prediction model to analyze clinical and laboratory patients’ radiotherapy response data by using a machine learning algorithm. The whole coclinical experiment involved 33 patients with diagnosed mid-to-low RC, and the machine prediction accuracy could achieve over 89% (48). The other study from Ting et al. displayed that the sensitivity, specificity, accuracy, and positive and negative predictive value of the PDO model for predicting chemotherapy drug responses were 63.33%, 94.12%, 79.69%, 90.48%, and 74.42%, which indicated that PDO responses consisted of the sensitivity in the respective patient in the clinic, which indicates that PDTOs can serve as a prediction model to guide the individualized selection of chemotherapy regimens for patients with RC (38). The work from another group identifies acid ceramidase (AC) as a target for improving radiotherapy treatment with the finding that PDOs presenting low AC expression were more radiosensitive than other models possessing higher levels of AC. However, the sample size of this study was small, and they did not demonstrate the original sample collection location of the organoid cultures (56). Another group investigated the radiosensitivity of CRC with PDOs of both colon and rectal tumors and focused on how the radiosensitivity of 13 RC PDOs correlated with clinical treatment outcomes. A significant mutual correlation is seen between the D0 (from the single hit multitarget algorithm, which is a single value to evaluate tissue radiosensitivity) of primary tumor PDO and the clinical response to neoadjuvant therapy (45). In a nutshell, the prediction accuracy could reach a level of 60%–90%, demonstrating the potential of RC PDOs to correctly identify clinical therapy responders and nonresponders and guide personalized therapy selection for RC patients.

In a newly published study, PDOs were very helpful in finding a new resistance mechanism of iCAFs and IL1α in RC patients. These PDOs were generated from biopsies collected prior to CRT from (non-pCRs) patients with poor along with fine prognoses and (pCRs) patients with pathological complete responses, and subsequently, these organoids detected different IL-1ra expression levels but comparable IL-1α expression. This group also created a preclinical RC mouse model (APTKA) through orthotopically transplanting organoids into C57BL/6 mice. Through a series of experiments, they identified the therapy-resistant capacity of iCAFs in RC and proposed the IL-1 pathway as a potential target for matrix repolarization and prevention for cancer-associated fibroblast senescence (43). Based on the previous achievement, this group recently further started an ACO/ARO/AIO-21 phase I trial to test the IL-1 receptor antagonist (IL-1 RA) anakinra combining with CRT therapy for RC, which set up a great example for a translational application from bench to bedside (47).

According to the above research, PDOs show high accuracy in predicting therapy responses and guiding personalized strategies. There is a matter of modifying culture methods to achieve a stable growth rate and improve the prediction accuracy in drug tests and targeted therapy research, which enables the application of RC PDOs in clinical practice and trials.

## Investigation of intratumoral heterogeneity and tumor evolution

It is currently reported that PDOs facilitate the study of intratumoral heterogeneity and tumor evolution by analyzing the differences between PDOs from primary tissue and from the metastatic sites in a patient. Organoids derived from different sites in the same CRC patient possess different patterns and distinct responses to diverse therapies (26, 57, 58), whereas there are only rare reports about RC PDOs in this field.

One RC-related study from Ganesh et al. mentions the metastatic potential of an *in vivo* transplanted RC organoid model. They created a patient-derived orthotopic xenograft model with human tumoroids, which could reflect the actual process, including cancer initiation, invasion, and metastasis in the rectum. After 22 and 30 weeks post-transplant, these *in vivo* models turned into invasive adenocarcinoma, which is consistent with the stage I or II characteristics in human RC. They also found lung and liver metastases with infiltrating normal parenchyma by poorly differentiated carcinoma. It is worth mentioning the observation that metastases beginning from the endoluminal rectum shared the same appearance with the metastases found in the individual patients. Their success in the generation of a RC PDO endoluminal model paves the way forward in the field of tumor evolution research (39). This application of the PDO system again proves the potential and sheds light on the RC metastasis assays field.

## Studying the tumor microenvironment with PDOs

RC has a poor prognosis for both metastases and a higher risk of local recurrence than CC in CRC. Four different molecular CRC consensus subtypes (CMS1–S4) have recently been delineated according to their transcriptome profiles and cellular heterogeneity. The CMS4 type was identified as having the worst prognosis with a feature of an abundant mesenchymal signature (59). Therefore, the tumor microenvironment (TME) and, particularly, cancer-associated fibroblasts (CAFs) consisting of heterogeneous populations have great importance in CRC research.

*In vivo* models are familiar tools to study the interaction between cancer cells in the microenvironment, whereas organoids as a model *in vitro* make the interactions visible. With the purpose of overcoming the limitation that organoids do not harbor diverse cell types or complex tissue compositions, scientists developed a cocultivation method incubating human organoids with human CAFs in order to activate fibroblasts and promote the growth of desmoplastic stroma (60). Immune cells can also be successfully activated and possess killing capacity when cocultured with PDOs.

For instance, a cocultivation method is reported by a research group in which circulating tumor reactive T lymphocytes could be coincubated together with human CRC organoids and stimulated into active status (61, 62). By establishing these models, it is promising that we could assess the efficacy of immune therapy under a controlled environment and produce individual T cells for adoptive T cell transferring. Another study treated CRC organoids with chemoradiotherapy and examined the capability of tumor-infiltrating T lymphocytes within the culture (63). They checked the cytotoxic effects as well in the co-culture system containing RC organoids and infiltrating T lymphocytes and found that the killing ability of PDOs from patients with a good therapy response was stronger than in PDOs from nonresponders. In newly published research, Nicolas et al. cocultured the supernatants from low or high IL-1ra expression organoids together with matched human intestinal fibroblasts and found that supernatants with insufficient IL-1ra expression activated a series of pro-inflammatory gene expression profiles (43). Patient RC organoid application in the study of the TME is indeed a remarkable achievement. We expect to see more high-quality research using PDOs as a tool in RC microenvironment field.

## Cancer modeling by genetic engineering of organoids

It is reported that cancer genetic mutations can be generated in wild-type organoids through technology such as CRISPR/Cas9 to facilitate research in oncology pathway mutants, tumor origin, and invasion. To date, there are fewer reports about RC organoid application in this field, so we use the research of CRC organoids.

As for CRC metastasis research, it has underscored the need for engineered organoids and emphasized the importance of genetic composition (64, 65).

By using CRISPR/Cas9 technology, oncologists could establish both advanced cancer and early tumor organoids with various mutations. The Medema group generated sessile serrated adenoma (SSA) organoids with the BRAFV600E mutation by engineering normal colon organoids to ensure regular growth. They later put forward that the CRC mesenchymal subtype could originate by activating RAS signaling through TGFβ stimulation together with BRAFV600E alternation (66). Another group made use of the SSAs characteristic of Rspondin gene fusions and first established chromosomal rearrangements with CRISPR/Cas9 to dig the impact of Rspondin fusions (67). This study introduced the idea that CRISPR/Cas9 technology can also be applied to delete, insert, or translocate chromosomes in addition to the use for mutating genes, which led the way to discover new therapy through targeting a specific pathway.

It is worth mentioning that Nicolas et al. activated *Il1a* transcription in irradiation-sensitive APTK organoids (Apc, Trp53, Tgfbr2, and K-rasG12D mutant) by CRISPR/Cas9 (43). Their data supports the conclusion that tumor cell–derived IL-1α polarizes CAFs toward an inflammatory phenotype, which then causes resistance to irradiation. A strong expression of inflammatory genes has been discovered in fibroblasts exposed to the fluid of APTK-sg*Il1a* organoids. APTK-sg*Il1a* organoids show an increased postirradiation stromal response with the features of suppressed CD8^+^ T cell infiltration along with increased Sirius red staining. However, irradiation did not further improve the level of macrophage and neutrophil infiltration with the IL-1α-dependent increase. Nicolas et al. combined CRISPR/Cas9 technology with RC organoids, enriching the field of study known as RC modeling.

In summary, CRISPR/Cas9 technology in combination with organoids suggests a potential strategy for basic cancer research and exploration in clinical application. We expect to see the development of this application in RC field.

## Discussion

In recent decades, organoid cultures of various types of tumors have been widely used in basic and translational cancer research, which is a major boost for the field. PDOs can truly reflect an individual’s condition and possess the capacity to predict individual responses to diverse clinical therapies, allowing clinicians to tailor treatment strategies for each patient and practice precision medicine. The establishment of living biological sample banks enables us to detect a wide range of molecular and different patterns of one cancer entity to further promote new medicine development and fully exploit existing drugs. The presented studies also reveal the possible applications in cocultivation assays with stromal, immune, or CAR cells, which may facilitate research in targeting stromal compositions to lead immune cells to their points.

However, current PDO systems do show some limitations, and there are gaps together with challenges between knowledge and the translational application of PDO models. First, culturing PDOs still costs a great deal, and the materials and culturing conditions used vary between laboratories. We present a summary in **Table 3** with methodologic differences and limitations of selected studies (**Table 3**). An agreement needs to be reached. Second, successful establishment of a PDO platform depends on many factors, such as the size of the biopsy samples, amount of living tumor cells, and cancer cell proliferation rate. Contamination and residue of other cell types could also be problems in the biopsy material. In addition, as for rectal cancer, successful PDOs biobanks are precious resources since most RC patients have received neoadjuvant therapy prior to surgical resection, which may affect its role as prognosis tool in preclinical and clinical practice. At the same time, the frequency of eligible organoids for RC entities, such as sarcomas, is far from 80%–90%, suggesting the existence of relevant subtype conditions that may require specific growth factors. The greatest current challenge in patient-derived RC organoid culture is the absence of a TME. PDOs cannot recapitulate the full structure and function (such as the ability to grow blood vessels) of primary tissue for lacking CAFs, stroma cells, and immune cells in their culture system. Breakthroughs are being made in this field with continuous improvement of existing culture methods (e.g., an air–liquid interface PDO model enabling coculture with endogenous tumor-infiltrating lymphocytes). Finally, in view of inter- and intratumor heterogeneity, PDOs may need more *in vivo* model practice compared with PDX. Thus, the RC PDO model used in precision medicine requires extensive optimization and more focused research along with prospective study.

**Table 3:** Methodologic differences and limitations of selected studies.

Reference	Methods used for isolation	Composition of the extracelluar matrix	Type and number of cells seeded	Media and growth factors used	Purity of cell composition of the organoid
(42)	• Dissociating cells using 2 mg/ml collagenase I • Filtering with a 70 μm nylon cell strainer • Centrifuging at 250*g* for 5 min at room temperature	15 μl Matrigel Matrix	50,000 live cells	50% advanced Dulbecco's modified Eagle's medium (DMEM);50% L-WRN conditioned media; Both supplemented with: 20% fetal bovine serum (FBS), 2 mM GLUTamax, 100 units/ml penicillin, 0.1 mg/ml streptomycin, 10 μM SB 431542(TGF-beta/Smad inhibitor), 10 μM Y-27632 (ROCK Inhibitor), 50 μg/ml gentamicin. Media were changed every other day.	Not mentioned
(43)	• Tumor biopsies were cut in small pieces, washed with PBS supplemented with 1x penicillin/streptomycin several times. • Incubating with 5mM PBS/EDTA for 10 min on ice with several vortex cycles • Incubating with PBS/EDTA 5 mM for 30 min on ice with several vortex cycles • Collecting the supernatant and filtering through 70 μm filters • Centrifuging at 800 rpm for 5 min	Matrigel polymerized at 37°C for 30 min	Not mentioned	Advanced DMEM F12 supplemented with: 1x Glutamax, 1x HEPES, 1x Penicillin/Streptomycin, 2.5% B27 supplement, 20% R-spondin, 10% Noggin condition media, 50 ng/ml human EGF, 2 mM N-Acetylcysteine, 500 nM TGF-β inhibitor A-83-01, 10 μM p38 MAPK inhibitor SB202190. 1000 μl media was added to each well (12 wells suspension plate)	Not mentioned
(45)	• Tumor tissues were incubated with digestion buffer (Advanced DMEM/F12 medium with 2% FBS, 100 U/mL penicillin/streptomycin, 500 U/mL collagenase, and 125 μg/mL Dispase type II) for 30 minutes at 37°C, shaking every 5 minutes. • Samples were washed with 10 mL of ADF-FBS medium and spun at 300 × *g* for 5 minutes.	40 ul Matrigel cell suspension per well overlaid with 500 μL of EN medium in 24-well culture plates.	Not mentioned	ADF basal medium: Advanced DMEM/F12 medium plus 1 mmol/L GlutaMAX, 1 mmol/L HEPES, and 100 U/mL penicillin/streptomycin. EN medium for tumor PDOs: ADF medium was supplemented with 10% Noggin conditioned medium (conditioned media were collected from cultures of HEK293 cells expressing recombinant Noggin proteins), 10 nmol/L gastrin I, 500 nmol/L A83–01, 10 mmol/L SB202190, 10 mmol/L nicotinamide, 1X B27 supplement, 1X N2 supplement, 1 mmol/L N-acetyl cysteine, and 50 ng/mL human recombinant EGF.	Not mentioned
(38)	• Tumor tissues were washed with 10 mL of Hanks Balanced Salt Solution containing antibiotics, minced with scissors. • Digesting with 5 mL of 5 mg/mL collagenase type II in Advanced DMEM/F12 for ≈4 h at 37 °C with gentle shaking and intermittent pipetting. • Samples were filtered with a 70 μm cell strainer and centrifuged at 300*g.* • Red blood cells were lysed using lysis buffer for 5 minutes.	Incubated with 30 ul Matrigel basement membrane matrix and polymerizing at 37°C for 30 min	For drug response analyses, organoids were resuspended in 2% Matrigel/organoid culture medium with 200–1000 clusters per milliliter	Advanced DMEM/F12 without other factors mentioned	Not mentioned about cell position. Success rate:69.77% in the pilot study;80.21% in the blinded study
(48)	• Tumor tissues were incubated with collagenase type II, dispase type II and Y-27632 for 30 min at 37°C. • Isolated cells were washed with PBS and centrifuged at 300 × g for 3 min at room temperature.	Matrigel (growth factor reduced, phenol red free)	Not mentioned	1× B27 supplemented with: 1.25 mM *N*-acetyl cysteine, 50 ng/ml human epidermal growth factor, 50 ng/ml human Noggin, 10 nM gastrin, 500 nM A83-01 and 100 mg/ml primocin, CHIR99021, R-spondin1. 10 uM Y-27632 was added to the culture medium to prevent anoikis.	PDTOs differentiated into enterocytes, goblet cells, and enterochromaffin cells and contained amplifying cells. Success rate:70%
(41)	• Tumor pieces were digested in mixed medium consisted of advanced DMEM/F12 with 2% FBS, Pen/Strep, 100 U/mL collagenase type XI, and 125 μg/mL dispose type II at 37°C for 40 min. • Adding TrypLE Express and DNase I medium for further digestion for 10 min • Samples were filtered through a 70 μm cell strainer, and centrifuged at 300 × g for 5 min.	Matrigel without mentioned about the dosage	Not mentioned	Used the same protocol described in Yao et al. study (16)	Not mentioned
(49)	• Tumor tissues were incubated in collagenase type II, dispase type II and Y-27632 for 30 min at 37°C	Matrigel on ice (growth factor reduced, phenol red free)	2 mm^3^-sized tumor piece was implanted into Central Institute for Experimental Animals NOG mice. Not mentioned about the type and number of cells seeded *in vitro*.	1xB27 supplemented with: 1.25 mM N-acetyl cysteine, 50 ng/mL human epidermal growth factor, 50 ng/mL human Noggin, 10 nM gastrin, 500 nM A83-01 and 100 mg/mL primocin. During the first 2–3 days, 10 uM Y-27632 was added to the culture medium to prevent anoikis.	Not mentioned
(51)	• Tumor specimens (2.5-6 × 7 mm in size) were minced into 0.1- 0.5 mm fragments, washed with ice-cold basal culture media. • Straining with a 70 µm pore mesh, and collected by centrifugation at 400*g* 4°C for 5 min.	25 µl drops Matrigel (Growth Factor Reduced) overlaid with 3 ml organoid growth media in pre-warmed 6-well tissue culture plates	Not mentioned	1xB27 supplemented with: 10 nM [Leu15]-Gastrin I, 1 mM N-acetyl-l-cysteine, 50 ng/mL for EGF, 100 ng/mL for Noggin, 500 nM for TGF-β receptor type I inhibitor A83-01 and 10 µM for p38 MAP kinase inhibitor SB202190. Organoid growth media without Y-27632 was refreshed every two to four days.	Not mentioned
(16)	• Tumor tissues were washed in the cold PBS with penicillin/streptomycin for 5 × 5 minutes, and then minced into tiny fragments. • Tissue fragments were digested in 8 mL digestion medium containing 7 mL DMEM medium, 500 U/mL collagenase IV, 1.5 mg/mL collagenase II, 20 μg/mL hyaluronidase, 0.1 mg/mL dispase type II, 10 μM RHOK inhibitor ly27632 and 1% fetal bovine serum on an orbital shaker at 37°C for 30-60 minutes. • Tumor cells were collected after centrifugation at 300-500 g for 5 minutes.	Incubated with Matrigel and polymerizing at 37°C, 5% CO_2_ for 5-8 min.	For irradiation response and drug tests, organoids were seeded in 48-well plate and density was adjusted to 10-15/μL Matrigel before seeding. Every well contained about 200 ± 50 organoids in 15 μL Matrigel with 300 μL culture medium. They applied organoid size (100 μm in diameter) to define right time for *in vivo* treatments.	Advanced DMEM/F12 medium, supplemented with: 500ng/mL R-spondin 1, 100ng/mL Noggin, 50ng/mL EGF, HEPES, Glutamax, Normocin, Gentamicin/amphoteritin B, N2, B27, n-Acetylcysteine, Niacinamide, Alk 4/5/7 inhibitor, p38 inhibitor, Gastrin and Prostaglandin E2.	Not mentioned about cell position. Success rate:77-85.7%
(54)	Tumor tissues were incubated with collagenase type II, dispase type II and Y-27632 for 30 min at 37°C.	Matrigel on ice (growth factor reduced, phenol red free)	For viability test 5,000 cells/10 µl Matrigel per well	1× B27, CHIR99021 supplemented with: R-spondin1, 1.25 mM *N*-acetyl cysteine, 50 ng/ml human epidermal growth factor, 50 ng/ml human Noggin, 10 nM gastrin, 500 nM A83-01 and 100 mg/ml primocin.	Not mentioned
(27)	• Tumor tissues were cut into <1 mm^3^ tumor fragments, washed with HBSS. • Samples were resuspended in Ammonium–Chloride–Potassium (ACK) Lysis Buffer to eliminate blood cells. • Digesting with 0.2 μ/ml of Liberase DH containing 10 μm Y-27632 for 60 min at 37°C. • Cells were filtered through a 250 μm sieve followed by a 100 μm cell strainer.	2 ml BME (reduced growth factor basement membrane extract) mixed with tumor cells. 40 μl droplets were seeded into a prewarmed six-well plate with seven droplets per well and incubated at 37°C for 10 min to solidify BME. Three milliliters of complete organoid culture medium were added to each well.	1 × 10^6^ tumor cells	Advanced DMEM/F12 supplemented with: 10 mM HEPES, 1× GlutaMAX and 1× penicillin/streptomycin, 500 nM A83-01, 1XB27 supplement, 50 ng/ml epidermal growth factor, 10 nM gastrin, 1 mM *N*-acetyl-l-cysteine, 10 mM nicotinamide, 10 nM prostaglandin E2, 6 μM SB20219 and 10 μM Y27632 Fresh medium was added every 3 days and PDTOs were passaged every 7 days.	100% cancer epithelial cellularity based on CDX2 and CK20 staining Pleomorphic single cells (*n* = 1), small solid cell clusters (*n* = 3), small patent glands (*n* = 1), medium patent glands (*n* = 1) and large cribriform glands (*n* = 3) Success rate:90%
(55)	• Tumor biopsies were washed in PBS and incubated with antibiotics for 30 min at RT. • Biopsies were cut into small pieces and digested with collagenase type IV (1 mg/mL in PBS for 30 min at 37 °C with continuous shaking in a water bath. • Disaggregating by passing the suspension through a 18 G needle and filtering through a 200-μm mesh into a 50 mL conical tube and centrifuged at 250× *g* for 5 min at 4°C.	Matrigel without mentioned about the dosage	Not mentioned	Advanced DMEM/F12 supplemented with: 10 mM HEPES, 10mM GlutaMAX and 1× N2, 1XB27 supplement,1:500 primocin, 1ug/ml gastrin, 0.1ug/ml Noggin, 1 mM *N*-acetyl-l-cysteine, 10 mM nicotinamide, 50ng/ml EGF, 0.02uM PGE2, 1 μM LY-2157299 and 10 μM Y27632 Culture medium was changed every second day.	Not mentioned about cell position. Success rate:55%
(56)	Not mentioned	40ul Matrigel	10000 cells	Intesticult™ Organoid Media supplemented with: 1% Penicillin-streptomycin and 10 μM Y27632 dihydrochloride, Rho kinase (ROCK) inhibitor	Not mentioned
(39)	• Tumor tissues were washed with ice-cold PBS-Abs buffer and chopped into 1 mm pieces in ice-cold PBS-DTT buffer. • Digesting with digestion medium (advanced DMEM/F12 with 2% FBS, Pen/Strep, 100 U/mL collagenase type XI, and 125 μg/mL dispase type II) at 37 °C for 40 min and further digested for 10 min by adding a half-volume of TrypLE Express, and 3 mg of DNase I per sample. • Samples derived from resected tumors were filtered through a 70 μm Cells Strainer, centrifuged at 300×g for 5 min.	Samples derived from biopsies were embedded in 800 μL Matrigel and samples derived from resected tumors were embedded in 1–2 mL of Matrigel. 50 μL/well overlaid with 500 μL culture medium after the Matrigel balls were polymerized. (24-well suspension plate)	Not mentioned	Advanced DMEM/F12 was supplemented with: antibiotic-antimycotic, 1×B27, 1×N2, 2 mM GlutaMAX, 10 nM gastrin I, 10 mM HEPES, 1 mM N-acetylcysteine, and 10 mM nicotinamide, 50% Wnt-3A conditioned medium, 20% R-spondin conditioned medium (media collected from HEK293 cell lines expressing recombinant Wnt3a and R-spondin1, kindly provided by Kevin P. O’Rourke and the S. Lowe laboratory), 100 ng/mL mouse recombinant noggin, 50 ng/mL human recombinant EGF, 500 nM A83–01, and 10 μM SB 202190. Upon expansion, RC PDOs were passaged and then cultured in medium without Wnt-3A, R-spondin, and noggin.	RC PDOs retained Alcian blue-positive and MUC-2-positive goblet cells, CK20 and CDX2-positive enterocytes, robust expression of E-cadherin (epithelial marker), and cytoplasmic/nuclear patterns. Success rate:77% (65/84)
(40)	• Chopped tissues were digested with 4 ml tissue digestion solution, 37°C water bath for 30 minutes; • Centrifuging at 1200 rpm and 4°C for 4 minutes, discarding the supernatant; • Suspending with Dulbecco's modified eagle medium/nutrient mixture F-12 (DMEM-F12), and filtering with 70 mu m and 40 mu m filters • Adding filtrate to low-adsorption dish, incubating for 1 hour in a 37°C and 5% carbon dioxide; • Centrifuging the liquid at 1200 rpm, 4°C for 4 minutes, discarding the supernatant; • Adding 1 ml phosphate buffered saline to wash, taking an appropriate amount and counting, and centrifuging the remaining portion at 1200 rpm and 4°C for 4 minutes, discarding the supernatant	50ul Matrigel (RTM: Gelatinous protein mixture) per well (48-well cell culture plate) solidifying for 20 minutes,overlaid with 500 ul preheated self-made culture medium to each well.	(2-4) *10^4 cells/ ul	Not mentioned Culture medium was changed every 3 days.	Not mentioned

New technology, such as genomic, transcriptomic, and proteomic analyses, are used on organoids and help us further understand tumor biology to define targeted treatment. It is promising to see that PDOs allow direct prediction of the individual response to identified RC therapies. In a nutshell, patient-derived cancer organoids show the potential to bridge the gap between the present basic research and clinical practice in the near future, but more detail is needed before we can really identify the areas of most impact in terms of research and clinical application.

## Methods

### Data sources and literature search strategy

This review followed the PRISMA guidelines. Two investigators (IC and YY) independently conducted a literature search using as combined keywords rectal cancer and organoid, patient-derived organoid on Pubmed (https://www.ncbi.nlm.nih.gov/pubmed/) and Web of Science (v. 5.35). The database search was run on published articles in the last two years, all languages, from database inception until May 20, 2022. In both databases, the following search strategy was used: terms were searched as follows: organoid AND rectal cancer; organoid AND rectal carcinoma; patient-derived organoid AND rectal cancer; patient-derived organoid AND rectal carcinoma. It is thought that these terms would identify the majority of manuscripts within a narrow definition of rectal cancer and organoid, patient-derived organoid, though it remains likely that relevant sections might be embedded within the methodology sections of particular projects and, thus, more challenging to identify. In addition, we conducted a search on ClinicalTrials (https://clinicaltrials.gov/) using the keywords colorectal cancer organoid or rectal cancer organoid.

### Study selection and data synthesis

All studies reporting information on RC and organoids, PDOs were included. One hundred twenty-four articles were identified and reviewed independently by two authors (IC and YY), and after all duplicates were removed, 41 articles were considered. After removing articles that were not in English and those that had simply a mention of the words with no further expansion, 21 articles were considered. Sixteen articles (of the 21) devoted a considerable amount of the manuscript to expand on those topics, and five articles (of the 21) are reviews or systematic reviews.

As for the search on ClinicalTrials, 24 studies were identified using the term “colorectal cancer organoid” and “rectal cancer organoid.” Eighteen studies were considered after all duplicates and studies unrelated to CRC or organoid were removed.

Thematic groupings and analyses were reviewed by an additional author (IC). All outcomes were included due to the wide range of use of the terminologies.

## Results

The manuscripts identified in this review (*n* = 21) followed six loosely defined thematic groups: a) analysis of the mutational landscape using high-throughput sequencing technologies (*n* = 7), b) drug screening to delineate novel treatment strategies (*n* = 4), c) personalized medicine based on the testing of individual PDOs (*n* = 10), d) investigation of intratumoral heterogeneity and tumor evolution (*n* = 1), e) studying the TME with PDOs (*n* = 2) and f) cancer modeling by genetic engineering of organoids (*n* = 2).

In terms of the search on ClinicalTrials (*n* =18), 13 studies (of the 18) were excluded for the following reasons: a) the study did not mention whether RC patients were involved in the studies (*n* = 10), b) studies related to CC PDO (*n* = 2), c) RC study used a malignant colonic organoid model (*n* = 1). Five studies associated with patient-derived RC organoid application were finally considered.

All publications or clinical studies that met the criteria were included in **Table 2**, and some of the publications and clinical studies excluded are shown in **Table 1** (28–37). They are presented subsequently in this order, reflecting the scientific continuum, moving from the characterization and acquisition of knowledge to the interpretation and finally toward clinical implementation.

## Author contributions

YY and IC conducted the review and applied the eligibility selection criteria for the identified manuscripts. IC validated the selected manuscripts and arbitrated any queries. CP participated in literature collecting. YY and IC wrote the manuscript. HW, XL and XW supervised the whole article. All authors contributed to the article and approved the submitted version.

## Funding

This study was supported by grants from the National Natural Science Foundation of China(82030099), the National Key R&D Program of China (2018YFC2000700), Shanghai Public Health System Construction Three-Year Action Plan(GWV-10.1-XK15), Innovative research team of high-level local universities in Shanghai.

## Acknowledgments

We thank all colleagues in the School of Public Health for their unconditional support.

## Conflict of interest

The authors declare that the research was conducted in the absence of any commercial or financial relationships that could be construed as a potential conflict of interest.

## Publisher’s note

All claims expressed in this article are solely those of the authors and do not necessarily represent those of their affiliated organizations, or those of the publisher, the editors and the reviewers. Any product that may be evaluated in this article, or claim that may be made by its manufacturer, is not guaranteed or endorsed by the publisher.
